# A Rare Case of Asymptomatic Aortic Dissection

**DOI:** 10.1016/j.jaccas.2025.104946

**Published:** 2025-08-13

**Authors:** Sanjana Murdande, Fowrooz Joolhar

**Affiliations:** Kern Medical Center, Bakersfield, California, USA

**Keywords:** aorta, dissection, echocardiography

## Abstract

**Background:**

Aortic dissection involves a tear leading to a false lumen. According to the Stanford classification, there are 2 main types—type A, involving the ascending aorta, and type B, exclusively involving the descending aorta. It is typically characterized by chest pain. However, some patients are asymptomatic and incidentally found with dissection.

**Case Summary:**

A 43-year-old patient presented to a primary care physician with palpitations. Transthoracic echocardiogram revealed thoracic aortic aneurysm with a possible type A aortic dissection. A computed tomography angiography of the aorta confirmed ascending type A aortic dissection to the isthmus. The patient refused emergent evaluation and left the hospital against medical advice twice. He remained asymptomatic until surgery.

**Discussion:**

Type A aortic dissections are life-threatening illnesses. Most patients present with tearing chest pain. Atypical presentations pose a diagnostic and therapeutic challenge.

**Take-Home Messages:**

Type A aortic dissection is a life-threatening illness with high mortality without an emergent surgery. Asymptomatic presentations increase morbidity and mortality.

## Graphical Abstract

**Figure:**
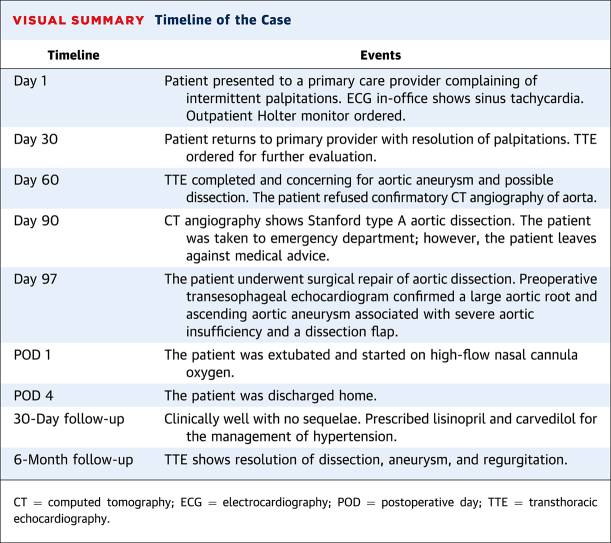

## History of Present Illness

The patient is a 43-year-old male with hypertension since age 15 and tobacco use of 50 pack years who presented to his primary care physician for routine appointment, during which he reported intermittent palpitations for 2 weeks. Vital signs in the clinic included a blood pressure of 142/64 mm Hg, heart rate of 113 beats per minute (bpm), respiratory of 18 breaths per minute, oxygen saturation of 97%, and temperature of 37 °C. Physical examination elucidated an obese male in no acute distress with a rapid heart rate on auscultation.

**Take-Home Messages**

• Aortic dissection is a life-threatening illness.
• Asymptomatic presentations increase morbidity and mortality, as patients can often go untreated.

## Past Medical History

The patient had a history of untreated hypertension from 15 to 42 years of age when he began treatment with lisinopril and nifedipine after establishing care with a primary physician in 2023. He also has a history of tobacco use of 50 pack years and continues to smoke.

## Differential Diagnosis

The differential diagnoses included arrhythmia, structural heart disease, including congestive heart failure, and noncardiac causes such as hyperthyroidism and anxiety.

## Investigations

Initial investigation with a primary care physician included same-day laboratory blood analysis and in-office electrocardiography (ECG). Results included a total cholesterol of 181 mg/dL, high-density lipoprotein of 31 mg/dL, triglycerides of 132 mg/dL, low-density lipoprotein of 124 mg/dL, hemoglobin of 18 g/dL, hematocrit of 52.7%, mean corpuscular volume (MCV) of 86, thyroid-stimulating hormone of 0.537, free T4 of 1.1, and hemoglobin A1_c_ of 5.2%. ECG in-office was that of sinus tachycardia with left ventricular hypertrophy and no conduction abnormalities.

Further investigation was with a Holter monitor, an ambulatory ECG device, which the patient wore for 2 days duration. This largely demonstrated sinus rhythm with an average heart rate of 100 beats/min. Upon return visit to primary care 1 month later, the patient reported that his palpitations had resolved. However, a formal transthoracic echocardiography (TTE) was ordered to evaluate for underlying structural heart disease, which was completed 1 month later. Transthoracic echocardiogram revealed thoracic aortic aneurysm, with the aortic root dilated to 6.5 cm and the ascending aorta to 6.2 cm, with evidence of possible dissection. Severe aortic regurgitation was also visualized. The patient was then referred to cardiology for further investigation, at which time a 2/6 diastolic murmur at the left lower sternal border was appreciated. No jugular venous distention, carotid bruits, focal neurologic, or pulse deficits were appreciated.

## Management

Following the disclosure of the TTE results, the patient refused emergent computed tomography angiography of aorta to confirm and evaluate the extent of the dissection for further treatment and surgical intervention. The patient left against medical advice. Computed tomography (CT) angiography of chest, abdomen, and pelvis was ordered emergently; however, it was eventually completed as an outpatient procedure approximately 1 month later. This demonstrated a Stanford type A aortic dissection with the aorta of maximum 7 cm, beginning at the sinus of Valsalva and terminating at the aortic isthmus. The dissection also involved the right brachiocephalic artery. He was promptly taken to the emergency department for immediate blood pressure control and emergent surgical treatment of the dissection. However, he left against medical advice prior to any therapy. Surgical repair in this patient was further delayed due to a lower-extremity abscess that required debridement and antibiotics. The patient eventually underwent valve-sparing surgical replacement of the aortic root, hemiarch replacement, aortic aneurysm repair, and aortic dissection repair almost 8 weeks from the initial diagnosis of aortic dissection on echocardiogram.

## Outcome and Follow-Up

The patient had total resolution of aortic regurgitation. Follow-up echocardiogram 6 months later confirmed the absence of regurgitation, aneurysm, and dissection.

## Discussion

The typical presentation of acute Stanford type A aortic dissection is that of tearing chest and/or back pain. Atypical presentations can include fatigue, dyspnea, and a variety of neurological presentations such as motor and sensory deficits related to the extension of the dissection. Acute type A aortic dissection is fatal if left untreated with 30-day mortality rate of up to 90% and an increase in mortality by 1% to 2% per hour during the first 24 to 48 hours.^1^ In a 2022 published cohort study of over 5,000 patients with acute type A aortic dissection from the International Registry of Acute Aortic Dissection, patients who underwent surgical treatment had a 48-hour mortality rate of 4.4% vs 23.7% in the medical management group.^2^ Given the time-dependent mortality of this condition, early and accurate diagnosis with imaging is critical to ensuring successful patient outcome. Asymptomatic presentations of type A aortic dissection are far less common and present a diagnostic challenge. With lower recognition, they are less likely to be successfully surgically managed, and prolonged duration without the optimal treatment increases the risk of mortality. In this patient with a high-risk physical exam finding of an aortic insufficiency murmur, CT angiography could have been pursued as an initial means of diagnostic evaluation according to the diagnostic algorithm for acute aortic dissection.^3^ CT imaging with contrast has a sensitivity and specificity over 95% for detecting acute aortic syndromes such as type A dissection, while TTE's suboptimal sensitivity does not make it the diagnostic test of choice.^3^ However, given that the patient was asymptomatic and the murmur was unrecognized on primary care evaluation, there was low clinical suspicion for acute aortic dissection, and the findings on TTE prompted further urgent clinical evaluation. Nonetheless, as the patient was delayed in obtaining his CT angiography, assessment with this imaging at the outset of presentation may have shortened the duration from diagnosis to management. There exist isolated reports of asymptomatic dissections that forego surgical therapy and avoid complications. In the case of Hattab et al,^4^ the patient's asymptomatic type A aortic dissection was solely medically managed due to patient refusal of surgery, and the patient remained stable at 5-month follow-up period. In the case of Song et al^5^, the patient refused surgery of her dissection, and follow-up CT chest 3 years later showed no significant changes. Nonetheless, surgery remains first-line therapy. Chronic type A aortic dissections are defined as those >1 month from the onset of symptoms and diagnosis.^6^ They have a lower early mortality than acute dissection of 6.1% vs 11.6%, respectively.^7^ This patient had an incidental finding of a very large aortic aneurysm on transthoracic echocardiogram and refused to have immediate further diagnostic workup; therefore, CT angiography and surgical repair were completed around 1 month later. The patient remained asymptomatic throughout this time. Given the prolonged duration of the dissection, this is assumed to be a chronic case. Similar cases of chronic hypertensive patients with asymptomatic Stanford type A aortic dissection were also found to have aortic aneurysms. In the case of Kumar et al^8^, a patient with chronic hypertension was found to have a thoracic aortic aneurysm with dissection and presented only with mild chest discomfort, eventually undergoing surgical repair. In the case of Tandan et al^9^, the patient was entirely asymptomatic and incidentally found to have a 6.5-cm thoracic aortic aneurysm with dissection, similarly undergoing surgical repair of aneurysm and dissection. It can be hypothesized that these patients and ours remained asymptomatic and without complication due to slow expansion of the aortic aneurysm and dissection leading to equalization of pressure within the true and false lumens.

## Conclusions

This case highlights a rare presentation of Stanford type A aortic dissection. The patient remained asymptomatic throughout diagnosis and management. Appropriate treatment with definitive surgical repair was delayed, however, effectively resolved the dissection once performed.

**Visual Summary:** Timeline of the Case

Timeline	Events
Day 1	Patient presented to a primary care provider complaining of intermittent palpitations. ECG in-office shows sinus tachycardia. Outpatient Holter monitor ordered.
Day 30	Patient returns to primary provider with resolution of palpitations. TTE ordered for further evaluation.
Day 60	TTE completed and concerning for aortic aneurysm and possible dissection. The patient refused confirmatory CT angiography of aorta.
Day 90	CT angiography shows Stanford type A aortic dissection. The patient was taken to emergency department; however, the patient leaves against medical advice.
Day 97	The patient underwent surgical repair of aortic dissection. Preoperative transesophageal echocardiogram confirmed a large aortic root and ascending aortic aneurysm associated with severe aortic insufficiency and a dissection flap.
POD 1	The patient was extubated and started on high-flow nasal cannula oxygen.
POD 4	The patient was discharged home.
30-Day follow-up	Clinically well with no sequelae. Prescribed lisinopril and carvedilol for the management of hypertension.
6-Month follow-up	TTE shows resolution of dissection, aneurysm, and regurgitation.

CT = computed tomography; ECG = electrocardiography; POD = postoperative day; TTE = transthoracic echocardiography.

**Equipment List:** Repair of Type A Aortic Dissection and Transverse Aortic Arch Replacement With Valve-Sparing Aortic Root Replacement and Repair of Aortic Root and Ascending Aortic Aneurysm

Imaging	• Transesophageal echocardiogram (TEE) (Philips Healthcare, USA)
Access	• Ultrasound machine (Philips Healthcare, USA) • Micropuncture needle and wire
Cardiopulmonary bypass	
Dissection repair and aortic arch replacement and valve-sparing aortic root replacement:	• 32-mm Gelweave graft (Terumo Aortic) • 34-mm Hemashield graft (Getinge) • Dual-stage venous cannula • Cross-clamp • Antegrade cardioplegia needle • Arterial cannula side port • Prolene suture running 4-0 • Teflon felt • Atrial and ventricular pacing wires • Chest tubes • Sternal wires

**Figure 1:**
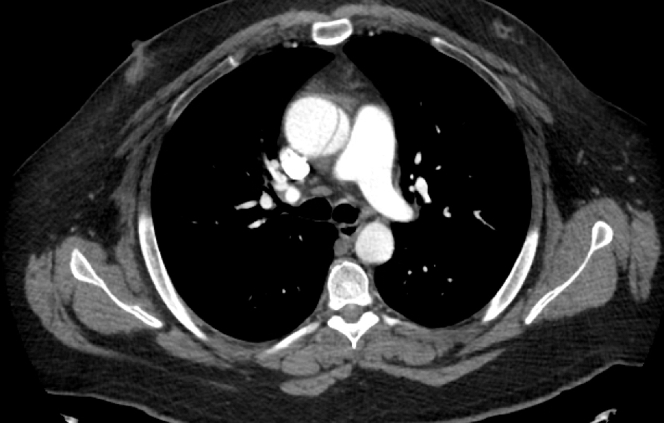
CT Angiography Chest, Abdomen, and Pelvis: Axial View CT angiography demonstrating ascending aortic dissecting aneurysm Stanford type A with visualization of the intimal flap, a hallmark sign. There is also involvement of the sinus of Valsalva where the aorta measures 7 cm, cardiomegaly with left ventricular dilatation, and dissection involvement of the right brachiocephalic artery. Contrast density is symmetric within the true and false lumen. CT = computed tomography.

**Figure 2:**
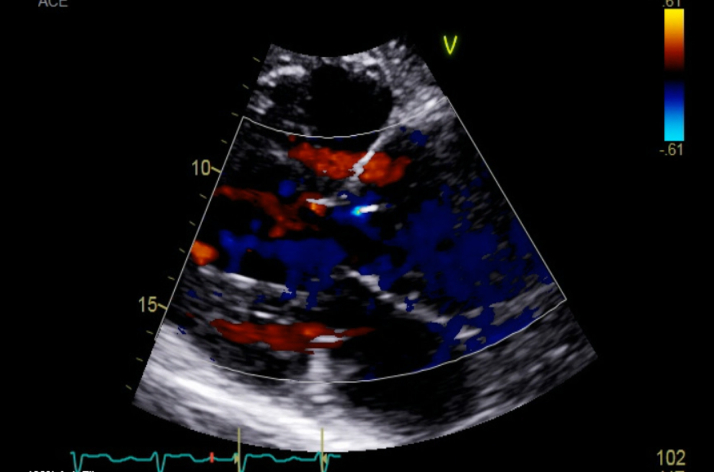
Transthoracic Echocardiogram: Parasternal Long Axis-View Color Doppler TTE demonstrating severe aortic regurgitation. Aortic valve leaflets are not well visualized. The aortic root is severely dilated measuring 6.5 cm. The ascending aorta is severely dilated measuring 6.2 cm. TTE = transthoracic echocardiography.

**Figure 3:**
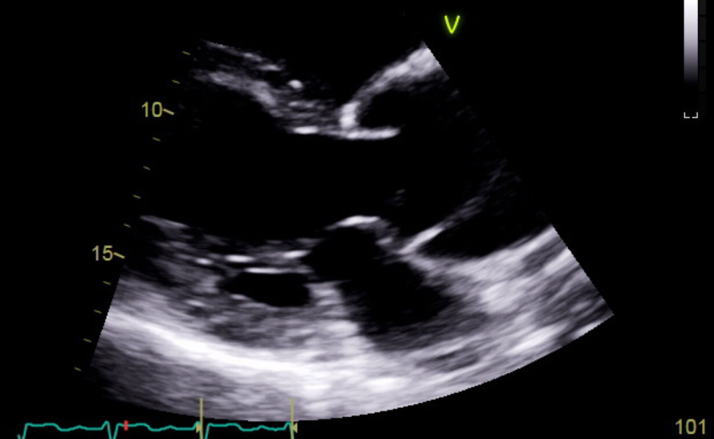
Transthoracic Echocardiogram: Parasternal Long-Axis View TTE demonstrating dissection flap in the ascending aorta, above the aortic valve leaflets. Dilation of ascending aorta is visualized as well. TTE = transthoracic echocardiography.

**Figure 4:**
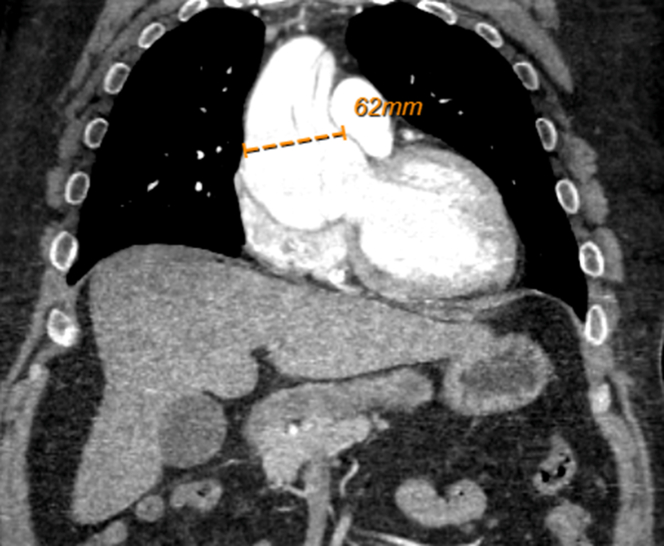
CT Angiography Chest, Abdomen, and Pelvis: Coronal View CT angiography demonstrating aortic aneurysmal dilatation of 6.2 cm.

## Funding Support and Author Disclosures

The authors have reported that they have no relationships relevant to the contents of this paper to disclose.
